# Additive impact of metabolic syndrome and sarcopenia on all-cause and cause-specific mortality: an analysis of NHANES

**DOI:** 10.3389/fendo.2024.1448395

**Published:** 2025-02-10

**Authors:** Meng Zhang, Qing-Yue Zeng, Linli Zhuang

**Affiliations:** ^1^ Department of Rheumatology and Immunology, West China Hospital of Sichuan University, Chengdu, Sichuan, China; ^2^ General Practice Ward/International Medical Center Ward, General Practice Medical Center, National Clinical Research Center for Geriatrics, West China Hospital, Sichuan University, Chengdu, Sichuan, China

**Keywords:** metabolic syndrome, sarcopenia, all-cause mortality, cardiovascular mortality, NHANES

## Abstract

**Background:**

Metabolic syndrome (MetS) and sarcopenia (SP) are increasingly significant public health issues in aging societies, sharing common pathophysiological mechanisms and being associated with severe health consequences. This study investigates the impact of MetS and SP on all-cause and cause-specific mortality using a longitudinal, nationally representative population-based cohort.

**Methods:**

The study analyzed data from the National Health and Nutrition Examination Survey (NHANES) conducted between 1999 and 2018. Mortality data were obtained from the National Death Index up to December 2019.

**Results:**

Among the 21,962 participants, 13,517 (61.5%) had neither MetS nor SP(MetS-/SP-), 5,407 (24.6%) had MetS only(MetS+/SP-), 2,698 (12.2%) had SP only(MetS-/SP+), and 340 (1.5%) had both MetS and SP(MetS+/SP+). Compared to the group without MetS and SP, the groups with MetS only, SP only, and both MetS and SP showed increased all-cause mortality, with adjusted hazard ratios (HR) of 1.23 (95% CI: 1.11-1.37), 1.63 (95% CI: 1.41-1.89), and 1.61 (95% CI: 1.33-1.95), respectively. The MetS+/SP+ group had the highest overall mortality risk (trend test p<0.0001). For cause-specific mortality, the MetS+/SP+ group exhibited increased cardiovascular mortality (HR: 1.89, 95% CI: 1.27-2.81), cardiac mortality (HR: 1.89, 95% CI: 1.25-2.86), respiratory mortality (HR: 2.63, 95% CI: 1.29-5.35), and diabetes mortality (HR: 8.79, 95% CI: 2.62-29.45) compared to the group without MetS and SP.

**Conclusion:**

The coexistence of MetS and SP significantly increases the risk of all-cause and cause-specific mortality. Individuals with either condition may require more vigilant management to prevent the onset of the other condition, thereby reducing mortality rates. These findings highlight the importance of integrated healthcare strategies targeting both MetS and SP to improve patient outcomes and longevity.

## 1 Introduction

Chronic diseases are increasingly placing a significant strain on global health. Metabolic Syndrome (MetS), characterized by central obesity, elevated triglycerides, reduced high-density lipoprotein cholesterol (HDL-C), hypertension, and elevated fasting glucose levels, has emerged as a major health concern (1). MetS is diagnosed when an individual exhibits three or more of these conditions. It has gained formal recognition from global health organizations, including the World Health Organization (WHO), and has been precisely defined through consensus by the International Diabetes Federation (IDF) and the American Heart Association/National Heart, Lung, and Blood Institute (AHA/NHLBI) (2, 3).

MetS is closely linked to insulin resistance, oxidative stress, and activation of the renin-angiotensin system, which not only underlie its pathogenesis but also have a detrimental impact on skeletal muscle health (4). These pathways are also implicated in sarcopenia (SP), a skeletal muscle disorder predominantly observed in the elderly, which manifests through reduced muscle strength, mass, and functionality. The incidence of sarcopenia exhibits regional and age-related variations, affecting approximately 1% to 29% of individuals living within communities and 14% to 33% of those in long-term care facilities (5). Sarcopenia significantly contributes to adverse clinical outcomes, including an increased risk of falls and fractures, the onset of physical disabilities, greater dependency on hospital services or a higher likelihood of hospitalization, decreased quality of life, and higher mortality rates. Furthermore, sarcopenia has strong connections with various chronic diseases, including cardiovascular diseases, respiratory diseases, and notably, MetS (6–10).

While MetS and SP are independently associated with elevated mortality risks, their potential interaction remains underexplored. MetS doubles the risk of cardiovascular mortality and increases all-cause mortality by 1.5 times (11). while sarcopenia similarly raises the risk of all-cause mortality, as evidenced in studies across diverse populations (12). Recent meta-analyses further confirm sarcopenia’s substantial association with mortality, irrespective of the population studied or the criteria for sarcopenia (13). Given their shared mechanisms—such as inflammation, oxidative stress, and metabolic dysregulation—it is plausible that the coexistence of MetS and SP may compound the risk of mortality. However, no longitudinal study has yet investigated the combined effects of MetS and SP on all-cause or cause-specific mortality.

This study aims to address this gap by leveraging data from the National Health and Nutrition Examination Survey (NHANES) to examine the cumulative impact of MetS and SP on mortality. Understanding their interaction could provide crucial insights into the mechanisms linking chronic conditions to adverse outcomes, thereby informing targeted prevention and management strategies.

## 2 Methods

### 2.1 Study design and population

**Figure 1:**
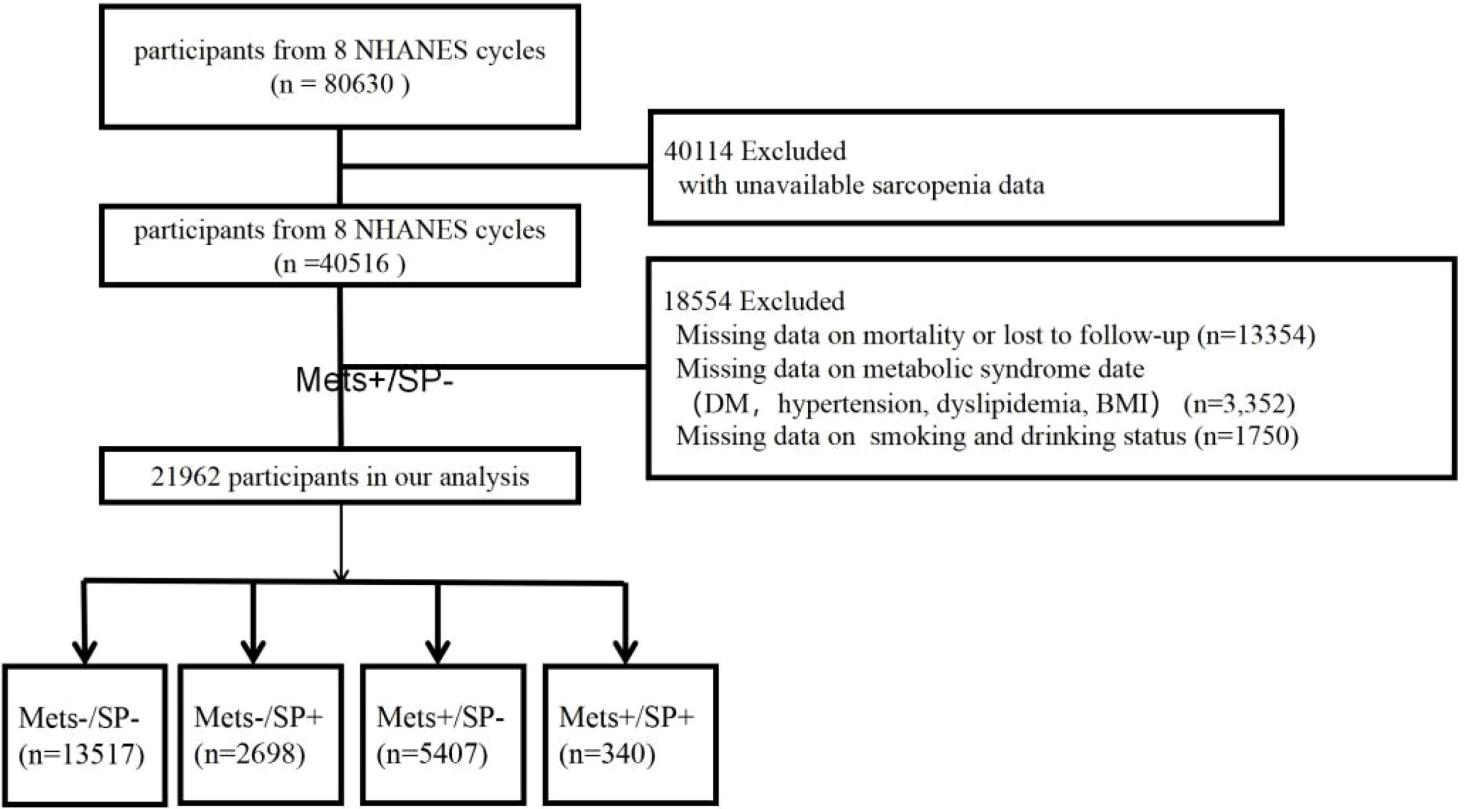
Flowchart of the selection strategy.

This study utilized data from the NHANES, which employs a complex, multistage probability sampling design to accurately represent the non-institutionalized civilian population of the United States. NHANES collects extensive health-related information through comprehensive interviews and physical examinations. The study protocol received ethical approval, and all participants provided written informed consent. The analysis included data from eight NHANES cycles conducted between 1999 and 2018.specifically the cycles for 1999–2000, 2001–2002, 2003–2004, 2005–2006, 2011–2012, 2013–2014, 2015–2016, and 2017–2018. These data were provided by the National Center for Health Statistics of the Centers for Disease Control and Prevention (CDC) and are detailed in the NHANES documentation files. The study population consisted of participants aged 20 years and older who met the following criteria: (1) availability of appendicular skeletal muscle mass and height measurements, and (2) availability of necessary data for diagnosing metabolic syndrome and mortality tracking. Exclusion criteria included individuals weighing more than 136 kilograms or taller than 192 centimeters due to DXA table limitations, pregnant women for safety reasons, and those recently exposed to contrast agents or radiation therapy to ensure scan accuracy. These criteria were implemented to ensure the accuracy and reliability of DXA measurements and to comply with NHANES safety protocols. The final dataset included 21,962 eligible participants with complete data on sarcopenia, metabolic syndrome diagnosis, and mortality, depicted in **Figure 1**.

### 2.2 Assessment of sarcopenia, metabolic syndrome and other covariates

Appendicular skeletal muscle mass (ASM) is a crucial indicator of skeletal muscle mass, representing the total lean mass of both arms and legs, measured using a DXA QDR-4500 Hologic scanner. Sarcopenia was primarily identified using the criteria established by the European Working Group on Sarcopenia in Older People 2 (EWGSOP2). The EWGSOP2 criteria were chosen due to their widespread acceptance in clinical and research settings, their focus on identifying individuals at risk of adverse outcomes, and their integration of muscle mass thresholds validated in diverse populations. These criteria define sarcopenia as a ratio of ASM to the square of an individual’s height, with thresholds set at ≤7.0 kg/m^2 for men and ≤5.5 kg/m^2 for women (14).For additional analysis, the Foundation for the National Institutes of Health (FNIH) criteria was employed, utilizing the ratio of ASM to body mass index (BMI), with cutoffs of ≤0.789 for men and ≤0.512 for women (15).

The diagnosis of MetS was based on the presence of three or more of the following conditions as per the National Cholesterol Education Program Adult Treatment Panel III (NCEP ATPIII) guidelines: central obesity, elevated fasting glucose, high serum triglycerides, low serum HDL-C, and hypertension (16).

Demographic, physical examination, and laboratory data were extracted from eight NHANES cycles. Demographic data included age, sex, and race. Education level was categorized into five tiers, and household income was classified into low, middle, and high based on the poverty income ratio. Marital status, smoking status, and alcohol consumption were defined according to specific standards (17). Diabetes mellitus (DM), hypertension, and cardiovascular disease (CVD) were determined through self-report, laboratory results, and medication use, with specific diagnostic criteria as follows: DM: Self-report, use of antidiabetic medication, fasting glucose ≥7.0 mmol/L, glucose ≥11.1 mmol/L in any test, or HbA1c ≥6.5% (18). Hypertension: Based on the 2017 AHA/ACC guidelines, an average of three resting blood pressure measurements with SBP ≥130 mmHg or DBP ≥80 mmHg, or self-reported diagnosis of hypertension (19). CVD: Identified through a medical questionnaire and self-reported physician diagnosis, including any positive indication of coronary heart disease, heart failure, myocardial infarction, angina, or stroke.

Physical examination and laboratory data included body mass index (BMI), waist circumference, total bilirubin, aspartate aminotransferase (AST), triglycerides (TG), total cholesterol, high-density lipoprotein cholesterol (HDL-C), creatinine, glycated hemoglobin (HbA1c), and urine albumin-to-creatinine ratio (UACR).

Based on these standards, study participants were categorized into four groups: (1) without MetS and SP (MetS-/SP-), (2) with MetS but without SP (MetS+/SP-), (3) with SP but without MetS (MetS-/SP+), and (4) with both MetS and SP (MetS+/SP+).

### 2.3 Study outcome

Mortality information was obtained from the National Death Index (NDI), which provided both the death status and cause of death. This data was linked with NHANES participant data using publicly accessible NDI files, with follow-up extending until December 31, 2019. Follow-up time began on the date participants were examined at the NHANES Mobile Examination Center and continued until the date of death or the end of the mortality follow-up period. Causes of death were classified according to the International Classification of Diseases, 10th Revision (ICD-10). The primary outcomes of the study were all-cause mortality and four cause-specific mortalities: cardiovascular disease mortality (codes I00-I09, I11, I13, I20-I51, and I60-I69), cardiac disease mortality (codes I00-I09, I11, I13, and I20-I51), respiratory disease mortality (codes J09-J18 and J40-J47), and diabetes mortality (codes E10-E14).

### 2.4 Statistical analysis

Following NHANES analytical guidelines, our statistical analysis incorporated the complex survey design and associated sampling weights. Participants’ demographic and clinical characteristics were summarized using weighted means and standard errors for continuous variables and counts and weighted percentages for categorical variables. Continuous variables were compared using weighted linear regression analysis, while categorical variables were analyzed using design-adjusted chi-square tests.

Cox proportional hazards models were employed to calculate the hazard ratios (HR) and 95% confidence intervals (CI) for the impact of MetS and SP on mortality. The proportional hazards assumption of the Cox models was evaluated using Schoenfeld residuals. Covariates were selected based on their established association with mortality and potential confounding effects identified from prior research. Model 1: Adjusted for age and sex. Model 2: Further adjusted for race/ethnicity, education level, household poverty income ratio, and marital status. Model 3: Additionally adjusted for BMI, waist circumference, total bilirubin, AST, TG, total cholesterol, HDL cholesterol, HbA1c, UACR, creatinine, alcohol consumption, and smoking status.

Interaction and subgroup analyses for all-cause and cause-specific mortality were conducted using Cox proportional hazards regression models, stratified by age (threshold of 40 years), sex, diabetes (DM), hypertension, and cardiovascular disease (CVD). These factors were selected based on their biological and epidemiological significance, as they may modify the combined impact of MetS and SP on mortality. The subgroup analyses were structured according to the specifications of Model 3 to ensure consistency and comparability across analyses. To enhance the robustness of our results, three sensitivity analyses were performed using the multivariable-adjusted Cox proportional hazards regression model. These included: (1) excluding participants who died within two years to minimize the potential for reverse causation, (2) excluding participants with pre-existing CVD to reduce confounding effects from advanced disease states, and (3) excluding individuals under 40 years of age to focus on an older population where MetS and SP are more prevalent.

Statistical significance was set at p < 0.05. All analyses were conducted using R software version 4.3.1 (R Foundation, Vienna, Austria).

## 3 Results

### 3.1 Baseline characteristics

Among the 21,962 participants, 13,517 (61.5%) had neither metabolic syndrome (MetS) nor sarcopenia (SP) (MetS-/SP-), 5,407 (24.6%) had MetS only (MetS+/SP-), 2,698 (12.2%) had SP only (MetS-/SP+), and 340 (1.5%) had both MetS and SP (MetS+/SP+). **Table 1** presents the baseline characteristics of these groups. Notably, the mean age of the MetS+/SP+ group was significantly higher compared to the other groups, being 39.54 years, 48.05 years, 43.49 years, and 59.64 years, respectively (p < 0.0001). Additionally, the MetS+/SP+ group had significantly higher levels of AST, HDL cholesterol, HbA1c, and UACR compared to the other groups (all p < 0.0001). Participants with both MetS and SP have lower household income, be predominantly non-Hispanic white, have an education level below 9th grade, and be current smokers compared to those without either condition.

**Table 1:** Baseline characteristics of the study participants.

Variable	Total (n=21962l	Mets-/SP- (n =l3517)	Mets-/SP+ !(n=2698)	Mets+/SP- !n=5407)	Mets+/SP+ (n=340)	Pvalue
Age, years, mean (SD)	42.21 (0.19)	39.54 (0.21)	43.49 (0.39)	48.05 (0.26)	59.64 (0.90)	< 0.0001
BMI, kg/m2, mean (SD)	28.00 (0.08)	27.55 (0.07)	21.29 (0.06)	32.94 (0.12)	24.32 (0.18)	< 0.0001
Waist circumference, cm, mean (SD)	95.95 (0.20)	93.88 (0.19)	81.40 (0.24)	109.38 (0.28)	95.11 (0.70)	< 0.0001
AST, U/L, mean (SD)	2S.ll (0.13)	24.72 (0.14)	24.24 (0.39)	26.54 (0.30)	26.85 (1.S4)	< 0.0001
Total bilirubin, µmol/L, mean (SD)	11.48 (0.08)	11.68 (0.08)	12.15 (0.17)	10.61 (0.10)	10.81 (0.36)	< 0.0001
Triglycerides, mmol/L, mean (SD)	1.67 (0.02)	1.34 (0.01)	1.20 (0.02)	2.78 (0.05)	2.67 (0.29)	< 0.0001
Total cholesterol, mmol/L, mean (SD)	S.09 (0.01)	5.01 (0.01)	4.99 (0.02)	S.38 (0.02)	5.37 (0.11)	< 0.0001
HDL-cholesterol, mmol/L, mean (SD)	1.36 (0.01)	1.43 (0.01)	1.55 (0.01)	1.09 (0.01)	1.22 (0.03)	< 0.0001
HbAlc, %,mean (SD)	S.47 (0.01)	S.31 (0.01)	S.30 (0.01)	S.96 (0.02)	5.99 (0.12)	< 0.0001
UACR, mg/g, median(Ql, Q3)	S.93 (4.03,10.33)	5.40 (3.81, 8.93)	6.97 (4.64,11.9S)	7.09 (4.S0,14.2S)	12.05 (6.18,22.73)	< 0.0001
Creatinine, µmol/L, median(Ql, Q3)	72.49 (61.88,88.40)	74.26 (61.90,88.40)	70.72 (61.88,79.56)	74.26 (61.88,88.40)	70.72 (61.88,97.20)	< 0.0001
Sex, n (%)						< 0.001
Female	10777 (49.8 1)	6376 (48.57)	1322 (52.60)	2907 (51.46)	172 (56.11)	
Male	11185 (50.19)	7141 (51.43)	1376 (47.40)	2500 (48.54)	168 (43.89)	
Ethnicity, n (%)						< 0.0001
Mexican American	4239 ( 8.75)	2457 (8.80)	463 (6.70)	1241(9.82)	78 (6.17)	
Non-Hispanic Black	4354 (10.35)	3174 (12.23)	222 (4.02)	950 (8.93)	8 (1.13)	
Non-Hispanic White	9627 (68.07)	5604 (66.24)	1410 (73.83)	2408 (69.51)	20S (80.2S)	
Other Hispanic	1530 ( 6.16)	985 (6.54)	132 (4.08)	391 (6.18)	22 (6.73)	
Other Race- Including Multi-Racial	2212 ( 6.67)	1297 ( 6.20)	471 (11.36)	417 ( 5.55)	27 ( 5.73)	
Educational level, n (%)						< 0.0001
9-llth Grade (Includes 12th grade with no diploma)	3101 (10.76)	1830 (10.04)	362 (10.80)	847 (12.35)	62 (17.75)	
College Graduate or above	5043 (28.52)	3409 (31.34)	695 (30.35)	885 (20.27)	54 (17.99)	
High School Grad/GED or Equivalent	5031 (23.84)	2993 (22.27)	618 (24.28)	1341 (27.71)	79 (28.81)	
Less Than 9th Grade	2281 ( 4.90)	1168 ( 4.25)	303 ( 5.06)	729 ( 6.24)	81 (12.29)	
Some College or AA degree	6506 (31.98)	4117 (32. 10)	720 (29.51)	1605 (33.43)	64 (23.16)	
Family income, n (%)						< 0.0001
High	7294 (43.46)	4738 (45.50)	863 (40.62)	1609 (39.84)	84 (32.44)	
Low	6445 (21.30)	3744 (20.11)	827 (22.70)	1754 (23.56)	120 (27.49)	
Medium	8223 (35.24)	5035 (34.39)	1008 (36.68)	2044 (36.60)	136 (40.07)	
Marital status, n (%)						< 0.0001
Living with partner	1860 { 8.57)	1296 (9.49)	186 (7.37)	364 (6.69)	14 (8.39)	
Married	11594 (55.45)	6874 (54.09)	1353 (50.98)	3167 (61.48)	200 (57.01)	
Never married	4625 (20.81)	3329 (23.31)	634 (25.59)	646 (12.06)	16 ( 7.06)	
other	3883 (15.18)	2018 (13.12)	525 (16.06)	1230 (19.78)	110 (27.55)	
Alcohol user, n (%)						< 0.0001
former	3233 (12.28)	1583 ( 9.76)	426 (12.33)	1113 (18.51)	111 (26.60)	
heavy	5040 (24.49)	3372 (26.17)	553 (22.52)	1074 (21.32)	41 (15.57)	
mild	7170 (34.05)	4538 (34 .57)	889 (34.41)	1644 (32.63)	99 (29.84)	
moderate	3568 (18.36)	2398( 19.76)	394 (17.91)	752 (15.02)	24 (12.11)	
never	2951 (10.83)	1626 ( 9.74)	436 (12.83)	824 (12.53)	65 (15.88)	
Smoking status, n (%)						< 0.0001
former	4777 (22.03)	2639 (20.26)	570 (21.03)	1442 (27.00)	126 (31.11)	
never	12060 (53.86)	7790 (56.87)	1377 (48.00)	2757 (49.48)	136 (36.40)	
now	5125 (24.11)	3088 (22.87)	751 (30.97)	1208 (23.53)	78 (32.49)	

BMI, body mass index; AST, aspartate aminotransferase; HDL-cholesterol, high density lipoprotein cholesterol; HbAlc, glycosylated hemoglobin; UACR, urinary albumin creatinine ratio.

### 3.2 All-cause and cause-specific mortality according to metabolic syndrome and sarcopenia status

**Figure 2:**
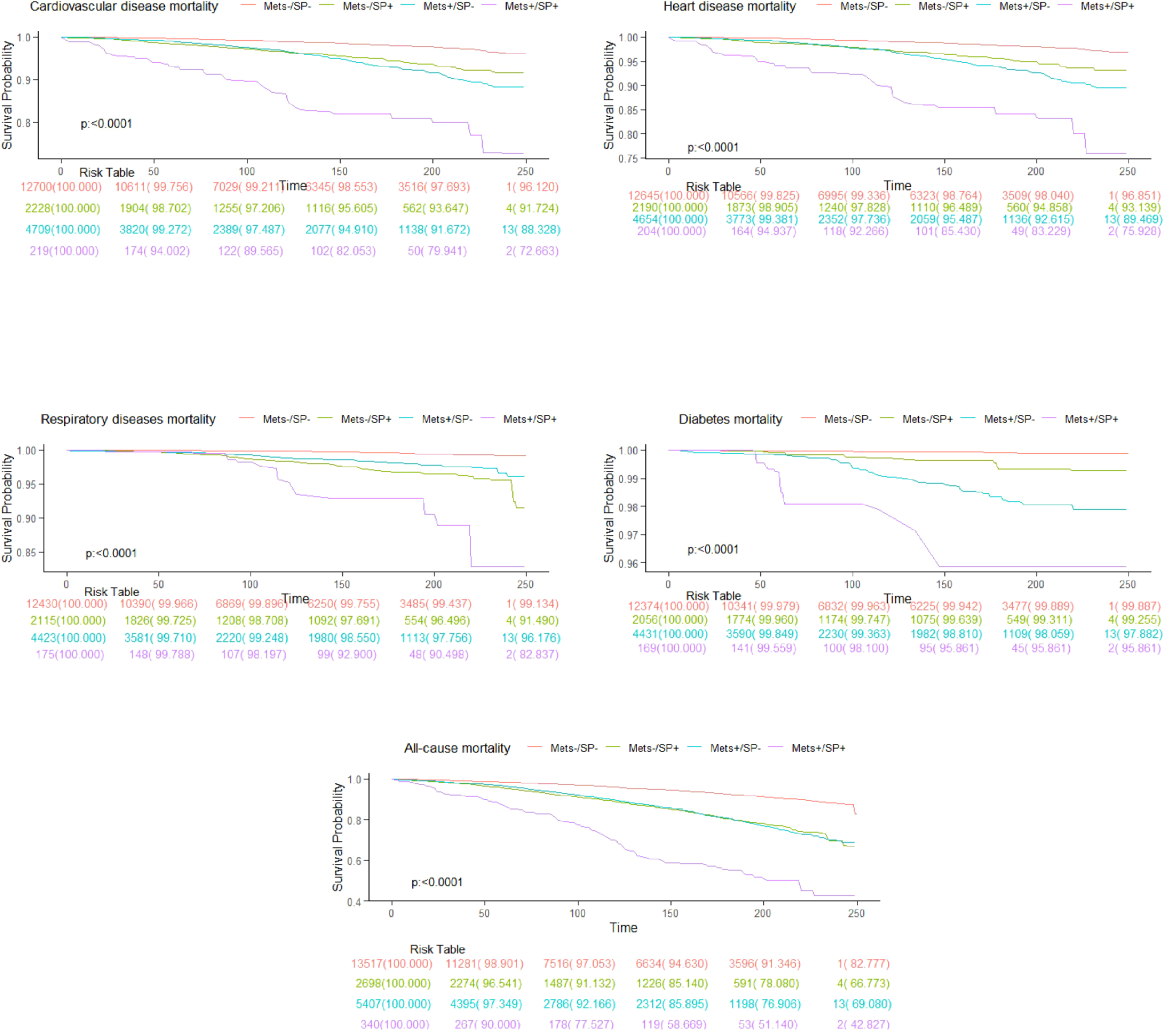
Kaplan-Meier curves show all-cause and cause-specific mortality.

During a median follow-up of 9.0 years (interquartile range: 5.3 to 16.6 years), there were a total of 3,047 deaths. Specific causes of death included 941 cardiovascular disease-related deaths, 778 heart disease deaths, 228 respiratory disease deaths, and 115 diabetes deaths. The overall mortality rates were 15.2 deaths per 1,000 person-years in the MetS-/SP- group, 22.4 in the MetS-/SP+ group, 19.8 in the MetS+/SP- group, and 28.1 in the MetS+/SP+ group. The survival curves for the four groups showed significant differences in overall and cause-specific survival rates (log-rank test p < 0.0001), with the MetS-/SP- group having the highest survival rates and the MetS+/SP+ group the lowest (**Figure 2**).

**Table 2** summarizes the HR for all-cause and cause-specific mortality based on MetS and SP status. Compared to the MetS-/SP- baseline group, the MetS-/SP+ group (HR = 1.63, 95% CI: 1.41–1.89, p < 0.0001), MetS+/SP- group (HR = 1.23, 95% CI: 1.11–1.37, p < 0.001), and MetS+/SP+ group (HR = 1.61, 95% CI: 1.33–1.95, p < 0.0001) showed increased risk of all-cause mortality. For cardiovascular disease mortality, the risks were also elevated in the MetS-/SP+ (HR = 1.78, 95% CI: 1.37–2.32, p < 0.0001), MetS+/SP- (HR = 1.40, 95% CI: 1.12–1.76, p = 0.004), and MetS+/SP+ (HR = 1.89, 95% CI: 1.27–2.81, p = 0.002) groups. The risk of heart disease mortality was higher in the MetS-/SP+ (HR = 1.79, 95% CI: 1.33–2.41, p < 0.001), MetS+/SP- (HR = 1.47, 95% CI: 1.15–1.88, p = 0.002), and MetS+/SP+ (HR = 1.89, 95% CI: 1.25–2.86, p = 0.003) groups. Respiratory disease mortality risk was also greater in the MetS-/SP+ (HR = 3.14, 95% CI: 1.89–5.21, p < 0.0001), MetS+/SP- (HR = 1.85, 95% CI: 1.21–2.82, p = 0.004), and MetS+/SP+ (HR = 2.63, 95% CI: 1.29–5.35, p = 0.01) groups. Diabetes mortality risk significantly increased in the MetS-/SP+ (HR = 3.26, 95% CI: 1.21–8.77, p = 0.02), MetS+/SP- (HR = 7.93, 95% CI: 3.27–19.24, p < 0.0001), and MetS+/SP+ (HR = 8.79, 95% CI: 2.62–29.45, p < 0.001) groups.

**Table 2:** Risks of all-cause and cause-specific mortality according to the presence of Mets or sarcopenia status.

group	All-cause mortality							
	crude model		Model 1		Model 2		Model 3	
	95%CI	p	95%CI	p	95%CI	p	95%CI	p
Mets-/SAR-	ref		ref		ref		ref	
Mets-/SAR+	2.81 (2.47,3.21)	<0.0001	1.67 (1.49,1.89)	<0.0001	1.65 ( 1.45,1.87)	<0.0001	1.63 {l.41,1.89)	<0.0001
Mets+/SAR-	2.80 (2.55,3.08)	<0.0001	1.52 {l.38,1.68)	<0.0001	1.44 ( l.30,1.59)	<0.0001	1.23 (1.11,1.37)	<0.001
Mets+/SAR+	7.68 (5.99,9.84)	<0.0001	2.07 {l.66,2.57)	<0.0001	1.92 (l.54,2.40)	<0.0001	1.61 (1.33,1.95)	<0.0001
p for trend		<0.0001		<0.0001		<0.0001		<0.0001

group	Cardiovascular disease mortality							
	crude model		Model 1		Model 2		Model 3	
	95%CI	p	95%CI	p	95%CI	p	95%CI	p
Mets-/SAR-	ref		ref		ref		ref	
Mets-/SAR+	2.82 (2.36, 3.38)	<0.0001	1.56 (l.29,1.89)	<0.0001	1.57 (1.28,1.93)	<0.0001	1.78 (l.37,2.32)	<0.0001
Mets+/SAR-	3.49 (2.95, 4.13)	<0.0001	1.72 (1.44,2.05)	<0.0001	1.65 (l.35,2.02)	<0.0001	1.40 (1.12,1.76)	0.004
Mets+/SAR+	10.24 {7.08,14.81)	<0.0001	2.26 (1.56,3.26)	<0.0001	2.29 (1.59,3.30)	<0.0001	1.89 (1.27,2.81)	0.002
p for trend		<0.0001		<0.0001		<0.0001		<0.0001

group	Heart diseases mortality							
	crude model		Model 1		Model 2		Model 3	
	95%CI	p	95%CI	p	95%CI	p	95%CI	p
Mets-/SAR-	ref		ref		ref		ref	
Mets-/SA R+	2.74 (2.18, 3.43)	<0.0001	1.54 (1.24,1.92)	<0.001	1.58 (l.26,1.97)	<0.0001	1.79 (l.33,2.41)	<0.001
Mets+/SAR-	3.74 (3.06, 4.57)	<0.0001	1.84 {1.49,2.27)	<0.0001	1.77 (1.40,2.23)	<0.0001	1.47 {l.15,1.88)	0.002
Mets+/SAR+	10.08 (6.53,15.56)	<0.0001	2.27 (1.50,3.45)	<0.001	2.31 (1.56,3.42)	<0.0001	1.89 (1.25,2.86)	0.003
p for trend		<0.0001		<0.0001		<0.0001		<0.0001

group	Respiratory diseases mortality							
	crude model		Model 1		Model 2		Model 3	
	95%CI	p	95%CI	p	95%CI	p	95%CI	p
Mets-/SAR-	ref		ref		ref		ref	
Mets-/SAR+	7.09 (4.58,10.98)	<0.0001	3.53 (2.29,5.42)	<0.0001	3.62 (2.36,5.57)	<0.0001	3.14 (1.89,5.21)	<0.0001
Mets+/SAR-	4.45 (2.89, 6.84)	<0.0001	1.98 (l.31,3.00)	0.001	1.77 (l.17,2.68)	0.01	1.85 (1.21,2.82)	0.004
Mets+/SAR+	19.44 {8.57,44.11)	<0.0001	3.86 (1.78,8.35)	<0.001	3.11 (1.49,6.49)	0.002	2.63 (1.29,5.35)	0.01
p for trend		<0.0001		<0.0001		<0.0001		<0.0001

group	Diabetes mortality							
	crude model		Model 1		Model 2		Model 3	
	95%CI	p	95%CI	p	95%CI	p	95%CI	p
Mets-/SAR-	ref		ref		ref		ref	
Mets-/SAR+	6.04 (2.38, 15.29)	<0.001	4.08 {l.54,10.83)	0.005	3.90 {l.51,10.03)	0.005	3.26 {l.21,8.77)	0.02
Mets+/SAR-	17.37 (8.50, 35.53)	<0.0001	8.78 (4.41,17.46)	<0.0001	8.25 (4.19,16.23)	<0.0001	7.93 (3.27,19.24)	<0.0001
Mets+/SAR+	42.10 (15.06,117.69)	<0.0001	11.12 (4.19,29.54)	<0.0001	9.92 (3.57,27.58)	<0.0001	8.79 (2.62,29.45)	<0.001
p for trend		<0.0001		<0.0001		<0.0001		<0.0001

crudel model: group

Model 1 was adjusted for age,sex.

Model 2 was adjusted for age, sex, race and ethnicity, educational level, family income and marital status.

Model 3 was adjusted for age, sex, race and ethnicity, educational level,family income.marital status, smoking status.alcohol user,body mass index, waist circumference,aspartate aminotransferase, total bilirubin,triglycerides,total cholesterol, high density lipoprotein cholesterol,glycosy lated hemoglobin, Creatinine, and urinary albumin creatinine ratio.

Using the FNIH criteria (ASM/BMI), **Supplementary Table S1** and **Supplementary Figure S1** show that individuals with MetS, SP, or both face higher risks of all-cause and cause-specific mortality compared to those without these conditions. Trend analysis indicated that HRs for all-cause and cause-specific mortality progressively increased from the MetS-/SP- group to the MetS+/SP+ group (trend test p < 0.0001).

### 3.3 Subgroup analyses and sensitivity analyses

Subgroup analyses demonstrated that the MetS+/SP+ group had consistently higher all-cause mortality risks across multiple subgroups, as summarized in **Table 3** and **Supplementary Tables S2A–S2D**. Among participants aged 41–85 years, the HR was 2.60, while no significant association was observed for those aged 20–40 years. Males and females in the MetS+/SP+ group showed HRs of 2.81 and 2.97, respectively. Participants with and without diabetes had HRs of 2.10 and 3.04, respectively. Similarly, participants with and without hypertension showed HRs of 2.17 and 2.54, while those with and without cardiovascular disease had HRs of 2.75 and 2.57, respectively.

**Table 3:** Subgroup of risks of all-cause mortality according to the presence of Mets or sarcopenia status.

Subgroup	Mets-/SP-	Mets-/SP+	p	Mets+/SP-	p	Mets+/SP+	p	p for trend	p for interactiond
AGE									0.008
41-85	ref	2.301 (1.966,2.693)	<0.0001	1.469 (1.300,1.661)	<0.0001	2.600 (2.111,3.204)	<0.0001	<0.0001	
20-40	ref	0.911 (0.527,1.573)	0.738	0.989 (0.585,1.672)	0.966	0.303 (0.034,2.655)	0.281	0.616	
sex									0.142
Male	ref	2.511 (2.106,2.993)	<0.0001	1.481 (1.220,1.797)	<0.0001	2.806 (2.005,3.926)	<0.0001	<0.0001	
Female	ref	2.030 (1.621,2.542)	<0.0001	1.788 (1.480,2.160)	<0.0001	2.965 (2.106,4.175)	<0.0001	<0.0001	
DM									0.434
no	ref	2.347 (1.979,2.784)	<0.0001	1.477 (1.275,1.712)	<0.0001	3.035 (2.311,3.985)	<0.0001	<0.0001	
yes	ref	1.964 (1.322,2.917)	<0.001	1.560 (1.171,2.079)	0.002	2.097 (1.206,3.644)	0.009	0.32	
Hypertension									0.001
yes	ref	2.190 (1.805,2.657)	<0.0001	1.266 (1.089,1.472)	0.002	2.136 (1.685,2.707)	<0.0001	<0.0001	
no	ref	2.219 (1.766,2.787)	<0.0001	1.525 (1.207,1.926)	<0.001	2.542 (1.471,4.392)	<0.001	<0.0001	
CVD									0.084
no	ref	2.230 (1.879,2.647)	<0.0001	1.493 (1.301,1.712)	<0.0001	2.568 (2.001,3.295)	<0.0001	<0.0001	
yes	ref	2.561 (1.974,3.323)	<0.0001	1.426 (1.125,1.809)	0.003	2.751 (1.803,4.198)	<0.0001	<0.0001	

DM, diabetes mellitus; CYD, cardiovascular disease; HR, hazard ratio; CI, confidence interval. Each stratification 1 as adjusted for race and ethnicity, educational level, family income, marital status, smoking status, alcohol user, body mass index. waist circumference, aspartate aminotransferase, total bilirubin, triglycerides, total cholesterol, high density lipoprotein cholesterol,glycosylated hemoglobin. Creatinine, and urinary albumin creatinine ratio.

In sensitivity analyses, after excluding participants who died within the first two years of follow-up, increased all-cause mortality risks were observed in the MetS-/SP+ (HR = 1.64), MetS+/SP- (HR = 1.25), and MetS+/SP+ (HR = 1.57) groups. Cardiovascular disease mortality risks were higher in the MetS-/SP+ (HR = 1.72), MetS+/SP- (HR = 1.39), and MetS+/SP+ (HR = 1.66) groups. Heart disease mortality risks increased in the MetS-/SP+ (HR = 1.72), MetS+/SP- (HR = 1.47), and MetS+/SP+ (HR = 1.68) groups. Respiratory disease mortality risks were elevated in the MetS-/SP+ (HR = 2.75), MetS+/SP- (HR = 1.71), and MetS+/SP+ (HR = 2.45) groups. Diabetes mortality risks significantly increased in the MetS-/SP+ (HR = 3.55), MetS+/SP- (HR = 8.82), and MetS+/SP+ (HR = 11.68) groups. After excluding participants under 40 years of age, the MetS-/SP+ (HR = 1.70), MetS+/SP- (HR = 1.28), and MetS+/SP+ (HR = 1.70) groups showed increased all-cause mortality risks. Cardiovascular disease mortality risks were higher in the MetS-/SP+ (HR = 1.87), MetS+/SP- (HR = 1.49), and MetS+/SP+ (HR = 1.92) groups. Heart disease mortality risks increased in the MetS-/SP+ (HR = 1.92), MetS+/SP- (HR = 1.57), and MetS+/SP+ (HR = 1.92) groups. Respiratory disease mortality risks were elevated in the MetS-/SP+ (HR = 3.17), MetS+/SP- (HR = 1.84), and MetS+/SP+ (HR = 2.67) groups. Diabetes mortality risks significantly increased in the MetS-/SP+ (HR = 4.64), MetS+/SP- (HR = 12.87), and MetS+/SP+ (HR = 14.34) groups. After excluding participants with a history of cardiovascular disease, increased all-cause mortality risks were observed in the MetS-/SP+ (HR = 1.58), MetS+/SP- (HR = 1.16), and MetS+/SP+ (HR = 1.41) groups. Cardiovascular disease mortality risks were higher in the MetS-/SP+ (HR = 1.81), MetS+/SP- (HR = 1.30), and MetS+/SP+ (HR = 1.74) groups. Heart disease mortality risk increased in the MetS-/SP+ (HR = 1.79) group but did not reach statistical significance in the MetS+/SP- and MetS+/SP+ groups. Respiratory disease mortality risks were higher in the MetS-/SP+ (HR = 2.23) and MetS+/SP+ (HR = 2.24) groups but did not reach statistical significance in the MetS+/SP- group. Diabetes mortality risks significantly increased in the MetS-/SP+ (HR = 4.79), MetS+/SP- (HR = 11.02), and MetS+/SP+ (HR = 4.27) groups (**Table 4**).

**Table 4:** Risks of all-cause and cause-specific mortality according to the presence of Mets or sarcopenia status without pre-existing CVD at baseline.

group	All-cause mortality							
	crude model		Model 1		Model 2		Model 3	
	95%CI	p	95%CI	p	95%CI	p	95%CI	p
Mets-/SAR-	ref		ref		ref		ref	
Mets-/SAR+	2.62 (2.25,3.05)	<0.0001	1.67 (1.46,1.91)	<0.0001	1.62 (1.40,1.87)	<0.0001	1.58 (1.33,1.86)	<0.0001
Mets+/SAR-	2.40 (2.15,2.68)	<0.0001	1.39 (1.24,1.57)	<0.0001	1.32 (1.18,1.48)	<0.0001	1.16 (1.02,1.31)	0.02
Mets+/SAR+	6.33 (4.70,8.53)	<0.0001	1.87 (1.42,2.46)	<0.0001	1.69 (1.29,2.22)	<0.001	1.41 (1.08,1.82)	0.01
p for trend		<0.0001		<0.0001		<0.0001		<0.0001

group	Cardiovascular disease mortality							
	crude model		Model 1		Model 2		Model 3	
	95%CI	p	95%CI	p	95%CI	p	95%CI	p
Mets-/SAR-	ref		ref		ref		ref	
Mets-/SAR+	2.77 (2.19, 3.50)	<0.0001	1.62 (1.28,2.05)	<0.0001	1.60 (1.25,2.06)	<0.001	1.81 (1.37,2.40)	<0.0001
Mets+/SAR-	3.01 (2.44, 3.72)	<0.0001	1.56 (1.26,1.92)	<0.0001	1.50 (1.21,1.86)	<0.001	1.30 (1.01,1.66)	0.04
Mets+/SAR+	7.95 (4.99,12.67)	<0.0001	1.99 (1.28,3.10)	0.002	1.97 (1.27,3.06)	0.003	1.74 (1.04,2.91)	0.03
p for trend		<0.0001		<0.0001		<0.0001		<0.0001

group	Heart diseases mortality							
	crude model		Model 1		Model 2		Model 3	
	95%CI	p	95%CI	p	95%CI	p	95%CI	p
Mets-/SAR-	ref		ref		ref		ref	
Mets-/SAR+	2.62 (1.96, 3.51)	<0.0001	1.56 (1.19,2.05)	0.001	1.59 (1.20,2.10)	0.001	1.79 (1.29,2 .48)	<0.001
Mets+/SAR-	3.07 (2.35,4.00)	<0.0001	1.60 (1.22,2.10)	<0.001	1.54 (1.17,2.03)	0.002	1.26 (0.98,1.62)	0.07
Mets+/SAR+	7.33 (4.29,12.54)	<0.0001	1.86 (1.14,3.03)	0.01	1.82 (1.15,2.88)	0.01	1.48 (0.92,2.39)	0.11
p for trend		<0.0001		<0.0001		<0.0001		<0.0001

group	Respiratory diseases mortality							
	crude model		Model 1		Model 2		Model 3	
	95%CI	p	95%CI	p	95%CI	p	95%CI	p
Mets-/SAR-	ref		ref		ref		ref	
Mets-/SAR+	6.64 (4.24,10.40)	<0.0001	3.21 (2.08,4.96)	<0.0001	3.17 (2.03, 4.94)	<0.0001	2.23 (1.20, 4.12)	0.01
Mets+/SAR-	3.65 (2.12, 6.27)	<0.0001	1.66 (0.98,2.82)	0.06	1.47 (0.88, 2.46)	0.14	1.67 (0.93, 2.98)	0.08
Mets+/SAR+	15.14 (6.42,35.70	<0.0001	2.84 (1.21,6.69)	0.02	2.26 (1.02, 5.02)	0.05	2.24 (1.00, 4.99)	0.05
p for trend		<0.0001		<0.0001		<0.0001		0.003

group	Diabetes mortality							
	crude model		Model 1		Model 2		Model 3	
	95%CI	p	95%CI	p	95%CI	p	95%CI	p
Mets-/SAR-	ref		ref		ref		ref	
Mets-/SAR+	8.96 (3.30,24.29)	<0.0001	6.65 (2.31,19.13)	<0.001	5.92 (2.02,17.34)	0.001	4.79 (1.58,14.47)	0.01
Mets+/SAR-	18.92 (8.79,40.73	<0.0001	10.83 (5.12,22.94	<0.0001	10.59 (5.08,22.09	<0.0001	11.02 (3.88,31.34	<0.0001
Mets+/SAR+	12.02 (3.46,41.83	<0.0001	3.78 (1.09,13.06)	0.04	3.20 (0.94,10.90)	0.06	4.27 1.01,18.03)	0.05
p for trend		<0.0001		<0.0001		<0.0001		<0.001

crudel model: group

Model l was adjusted for age, sex.

Model 2 was adju sted for age, sex, race and ethnicity, educational level, family income and marital status.

Model 3 was adju sted for age, sex, race and ethnicity, educat ional level, family income, marital status, smoking status.alcohol user, body mass index, waist circumference, aspartate aminotransferase, total bilirubin, tr iglycerides, total cholesterol, high density lipoprotein cholesterol, glycosy lated hemoglobin, Creatinine, and urinary albumin creatinine ratio.

**Supplementary Tables S3**, and **S4** as well as **Supplementary Figures S2**, **S3**, and **S4**, show the results of various models and Kaplan-Meier.

## 4 Discussion

MetS and SP are highly prevalent worldwide and represent a significant public health burden. This longitudinal study evaluated the impact of MetS and SP on all-cause mortality and cause-specific mortality, including cardiovascular, heart, respiratory, and diabetes-related mortality. The findings indicate that the coexistence of MetS and SP significantly increases the risk of all types of mortality (overall mortality HR = 1.61, cardiovascular mortality HR = 1.89, heart mortality HR = 1.89, respiratory mortality HR = 2.63, and diabetes mortality HR = 8.79). Additionally, the presence of either condition alone was also associated with increased risks of all-cause and cause-specific mortality.

Although the specifics are not fully understood, MetS and SP are considered to have close and complex interconnections. Several characteristics of MetS, including insulin resistance (IR), abnormal adipose tissue, and persistent chronic systemic inflammation that negatively impact muscle homeostasis, leading to reduced muscle mass and strength (1, 4, 20–23). In compensatory hyperinsulinemia due to IR, glycogenesis is poorly suppressed, protein degradation is accelerated, and protein synthesis is reduced (24). Abnormal adipose tissue, infiltrated by activated immune cells, results in heightened systemic inflammation and reduced production of key adipokines like leptin. This disruption impacts skeletal muscle, adversely (4, 25). Persistent inflammation, a key factor in the development of MetS, is also closely linked to sarcopenia. Elevated levels of proinflammatory cytokines like IL-6, CRP, and TNF-αhave been found to negatively impact muscle mass and function (26). Skeletal muscle plays a crucial role in insulin-induced glucose metabolism. Furthermore, the loss of muscle mass is closely associated with IR and MetS. Furthermore, myofibers in skeletal muscles can counteract metabolic issues by releasing proteins and myokines. Reduction in muscle mass, typically due to aging or diseases, exacerbates glucose metabolism problems (27). Based on these mechanisms, MetS and SP may each increase the risk of the other. In a study of 1,971 elderly Japanese community dwellers, the relationship between MetS and SP was explored. The findings showed a higher prevalence of sarcopenia in individuals with MetS, especially in men aged 65–74 years, with an odds ratio (OR) of 4.99 (28). Conversely, A meta-analysis encompassing 13 cross-sectional studies with 35,581 middle-aged and older non-obese adults revealed that 36.45% of individuals with sarcopenia also had MetS, indicating a positive correlation between MetS and sarcopenia (29). Decreased muscle mass can lead to metabolic syndrome (MetS), while increased muscle mass can help prevent it. In Kim et al.’s study of 13,620 people, a strong link was found between sarcopenia and MetS; a quartile increases in limb skeletal muscle index (SMI) reduced MetS risk by 56% (30). In a seven-year Korean study of 11,639 adults, 20.1% developed MetS, with those having higher baseline SMI showing significantly lower MetS rates than those with lower SMI (OR = 0.61) (31). MetS and SP detrimentally affect quality of life, leading to increased frailty, weakness, dependency, and higher morbidity and mortality rates. With the rapidly growing incidence and prevalence of both MetS and SP, their bidirectional relationship could pose amplified health risks in the increasingly aging society.

The results of this study indicate that participants with both MetS and SP have the highest risks of overall, cardiovascular, heart, respiratory, and diabetes-related mortality. These risks are higher than those associated with either MetS or SP alone, demonstrating a significant trend (**Table 2**). To our knowledge, this is the first nationwide population-based study that groups participants by MetS and SP status and compares mortality risks. We used both AWGS and FNIH criteria to assess low muscle mass, noting that HRs were lower when using FNIH criteria. The Kaplan-Meier survival curves (**Figure 2**) show significant differences in overall and cause-specific survival rates among the four groups. The MetS-/SP- group had the highest survival probabilities, indicating the lowest mortality risk, while the MetS+/SP+ group exhibited the steepest decline, highlighting the combined adverse impact of MetS and SP. The MetS+/SP- and MetS-/SP+ groups showed intermediate survival curves, reflecting the independent contributions of MetS and SP to increased mortality. The results remained consistent after excluding participants who died within two years, those with pre-existing CVD, or those under 40 years of age. Interestingly, in the sensitivity analysis of respiratory disease mortality after excluding participants with a history of CVD, the statistical significance for patients with MetS alone disappeared. Conversely, patients with SP alone and those with both MetS and SP continued to show an increased risk of respiratory disease mortality. When analyzing the impact of MetS on mortality without considering SP status (MetS+/SP- group), the risks for all-cause and four cause-specific mortalities were significantly elevated. Similarly, the group with SP alone (MetS-/SP+) also showed increased risks for all-cause and cause-specific mortalities.

This finding is consistent with recent research (11, 32). During a 7-year follow-up study involving 11,011 participants, sarcopenia was found to significantly increase the risk of CVD, stroke, and cardiac events (33). Additionally, studies in diabetic populations revealed that lower muscle mass, particularly in the arms, was associated with an elevated risk of both CVD and non-CVD mortality (34). A population-based prospective cohort study of 780 community-dwelling older adults found that sarcopenia significantly contributes to breathlessness in this population. Measuring sarcopenia in older adults may provide valuable opportunities for preventing age-related breathlessness (35). Additionally, other studies have shown that sarcopenia significantly impacts patients with chronic obstructive pulmonary disease, exacerbating their morbidity and mortality and suggesting that Dickkopf-3 could be a potential target for the diagnosis and treatment of sarcopenia in COPD patients (36, 37). MetS contributes to cardiovascular mortality through mechanisms like chronic inflammation, oxidative stress, endothelial dysfunction, and insulin resistance, which accelerate atherosclerosis (38). Sarcopenia, characterized by reduced muscle mass and strength, exacerbates metabolic dysfunction and increases vulnerability to cardiovascular complications (39). For respiratory mortality, the coexistence of MetS and SP likely amplifies risks through systemic inflammation, impaired respiratory muscle function, and reduced pulmonary reserve. Sarcopenia weakens respiratory muscles, increasing the risk of respiratory failure, while MetS compounds this with obesity-related hypoventilation and airway obstruction. The combined impact of MetS and SP likely creates a synergistic effect, leading to higher mortality risks than either condition alone (35, 40).

Further investigation may be necessary to explore the impact of MetS and SP on mortality across diverse ethnic groups. The rising older adult population could lead to more cases of MetS and SP, increasing the risk of cardiometabolic diseases and mortalities. Patients with both MetS and SP often exhibit stronger insulin resistance, inflammation, and vitamin D deficiency, which are linked to a higher risk of cardiometabolic issues, physical decline, and death (4). Given these findings, we recommend that the coexistence of sarcopenia and metabolic syndrome be recognized as an independent determinant of mortality risk in clinical assessments. Clinicians should routinely screen for both conditions, particularly in older adults, using validated diagnostic tools such as the EWGSOP2 criteria for sarcopenia and established MetS criteria. Early identification of at-risk individuals could enable timely interventions, such as targeted exercise programs, nutritional support, and pharmacological management of metabolic abnormalities and inflammation. These measures emphasize the importance of a multidisciplinary approach to managing MetS and sarcopenia, addressing both individual risk factors and broader population-level interventions to mitigate their combined impact on health and survival.

This study has several strengths and some limitations. strengths: Comprehensive clinical variables available; Large, representative U.S. population sample; Follow-up period of 9.0 years for mortality assessment; Robust results supported by sensitivity and subgroup analyses; Comprehensive assessment of mortality prognosis. Limitations: NHANES dataset lacks follow-up data on MetS and muscle-related factors, preventing assessment of longitudinal changes; Absence of muscle strength and function data in NHANES; future research should include these variables in longitudinal studies; Residual confounding factors might still affect the analysis despite adjustments.

## 5 Conclusion

In summary, the combination of MetS and SP as coexisting conditions significantly elevates the risk of mortality from various causes, including cardiovascular, heart, respiratory, and diabetes-related issues, beyond the risk associated with each condition individually. These results suggest a new strategy where vigilant monitoring and preventive measures against additional diseases are crucial in reducing the mortality risk in patients with either MetS or SP.

## Data Availability

The original contributions presented in the study are included in the article/**Supplementary Material**. Further inquiries can be directed to the corresponding author.
