# Supplementary material for: Additive impact of metabolic syndrome and sarcopenia on all-cause and cause-specific mortality: an analysis of NHANES
Source: Front Endocrinol (Lausanne). 2025 Feb 10;15:1448395. doi: 10.3389/fendo.2024.1448395 (PMC11847694; doi:10.3389/fendo.2024.1448395)
Supplement: Supplementary file 1 [file DataSheet1.docx]

**Additive impact of metabolic syndrome and sarcopenia on all-cause and cause-specific**

**mortality: A longitudinal nationwide population-based study**

[Figure S1.Kaplan-Meier curves show all-cause and cause-specific mortality, utilizing the FNIH criteria (ASM/BMI). 2](#_Toc88427334)

[Figure S2. Kaplan-Meier curves show all-cause and cause-specific mortality, excluding Participants who died within two years. 3](#_Toc88427335)

[Figure S3. Kaplan-Meier curves show all-cause and cause-specific mortality differences by SII, excluding participants under the age of 40. 4](#_Toc88427336)

[Figure S4. Kaplan-Meier curves show all-cause and cause-specific mortality differences by SII, excluding initial Cardiovascular diseases.](#_Toc88427336) 5

[Table S1. Risks of all-cause and cause-specific mortality according to the presence of Mets or sarcopenia status utilizing the FNIH criteria (ASM/BMI).](#_Toc88427341) 6

[Table S2A. Subgroup of Risks of cardiovascular diseases mortality according to the presence of Mets or sarcopenia status.](#_Toc88427342) 7

[Table S2B.Subgroup of Risks of heart diseases mortality according to the presence of Mets or sarcopenia status.](#_Toc88427343) 7

[Table S2C.Subgroup of Risks of respiratory diseases mortality according to the presence of Mets or sarcopenia status.](#_Toc88427344) 8

[Table S2D.Subgroup of Risks of diabetes mortality according to the presence of Mets or sarcopenia status.](#_Toc88427344) 8

[Table S3.Risks of all-cause and cause-specific mortality according to the presence of Mets or sarcopenia status without participants who died within two years.](#_Toc88427345) 9

[Table S4.Risks of all-cause and cause-specific mortality according to the presence of Mets or sarcopenia status without participants under the age of 40. 1](#_Toc88427345)0

[Table S5.Risks of all-cause and cause-specific mortality according to the presence of Mets or sarcopenia status without pre-existing CVD at baseline.](#_Toc88427345) 11


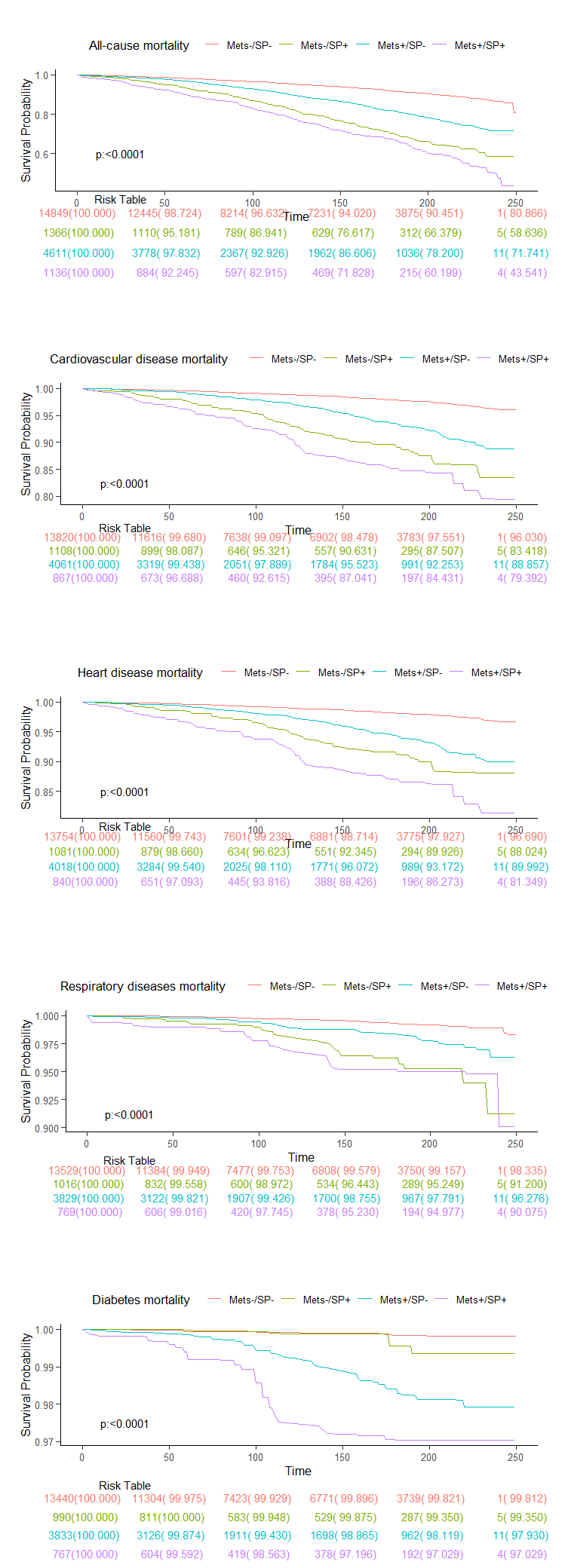


**Figure S1.**Kaplan-Meier curves show all-cause and cause-specific mortality, utilizing the FNIH criteria (ASM/BMI).


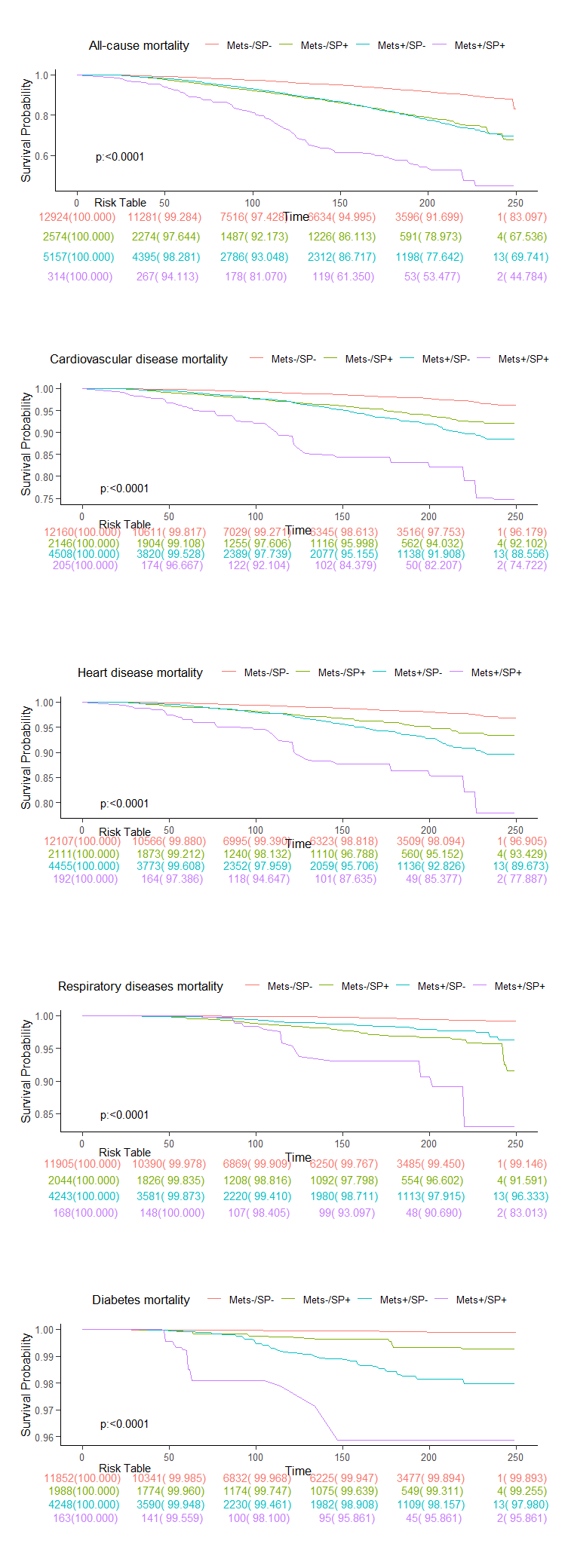


**Figure S2.** Kaplan-Meier curves show all-cause and cause-specific mortality, excluding Participants who died within two years.


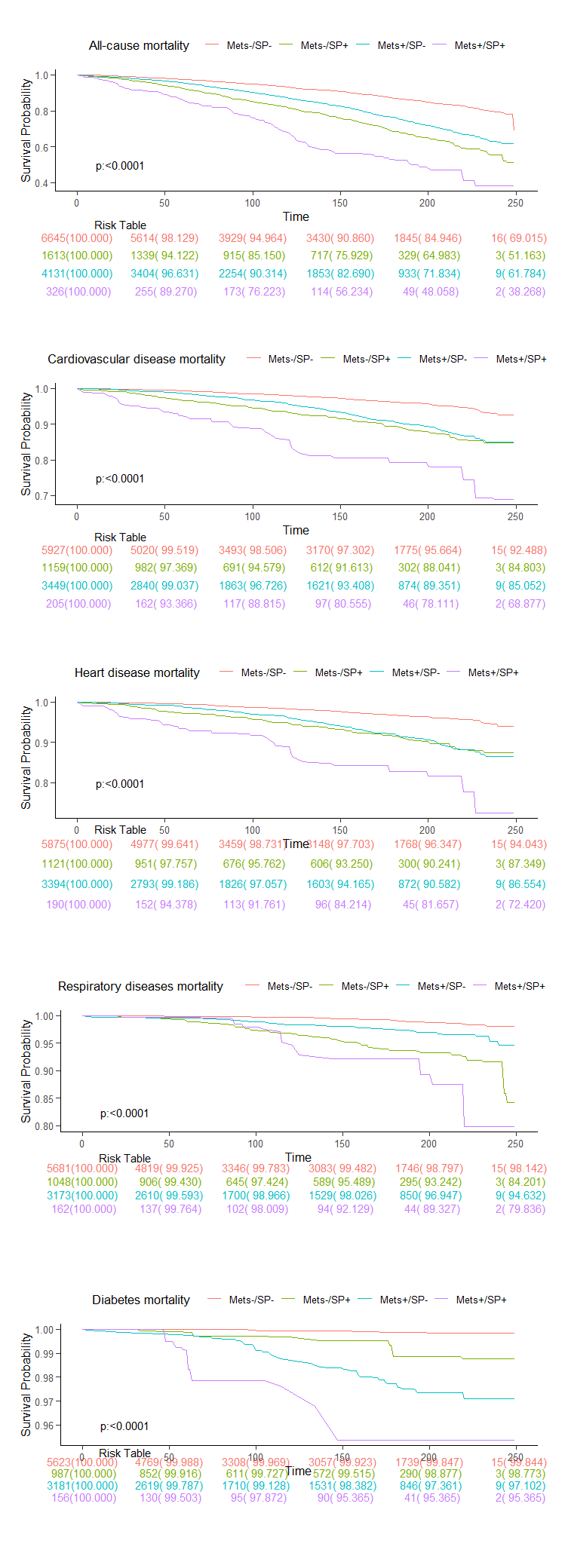


**Figure S3.** Kaplan-Meier curves show all-cause and cause-specific mortality differences by SII, excluding participants under the age of 40.


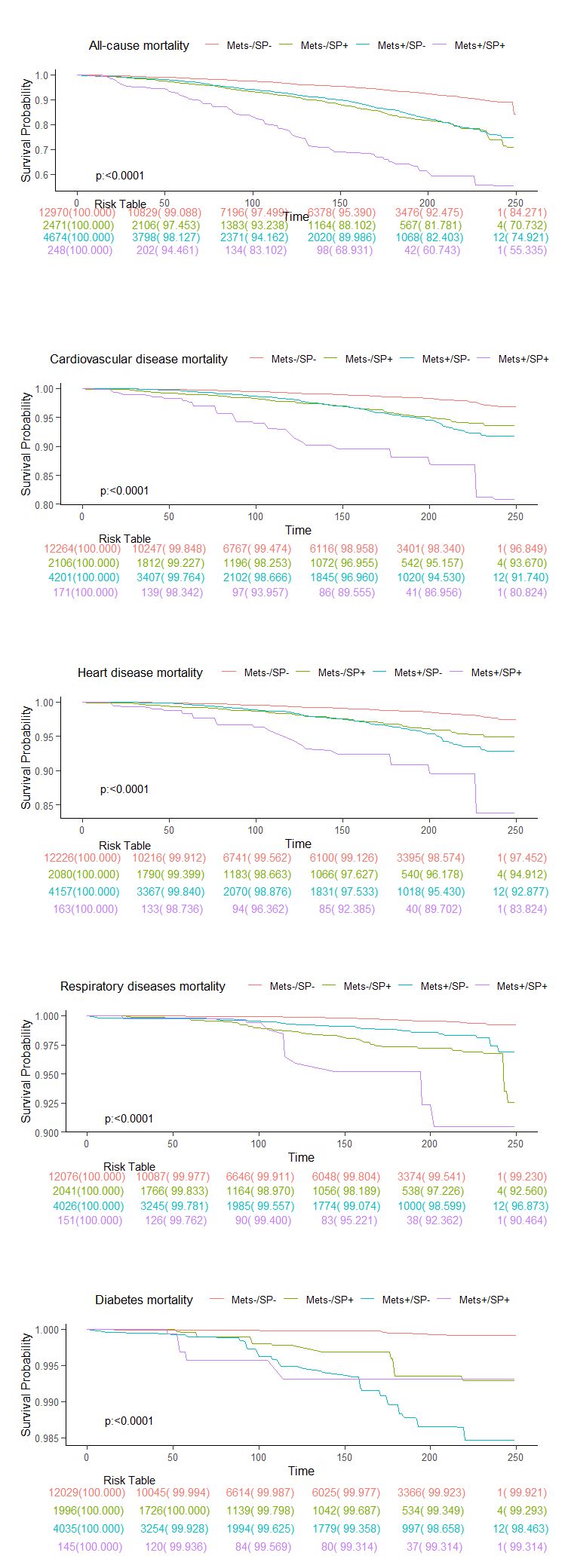


**Figure S4.** Kaplan-Meier curves show all-cause and cause-specific mortality differences by SII, excluding initial Cardiovascular diseases.


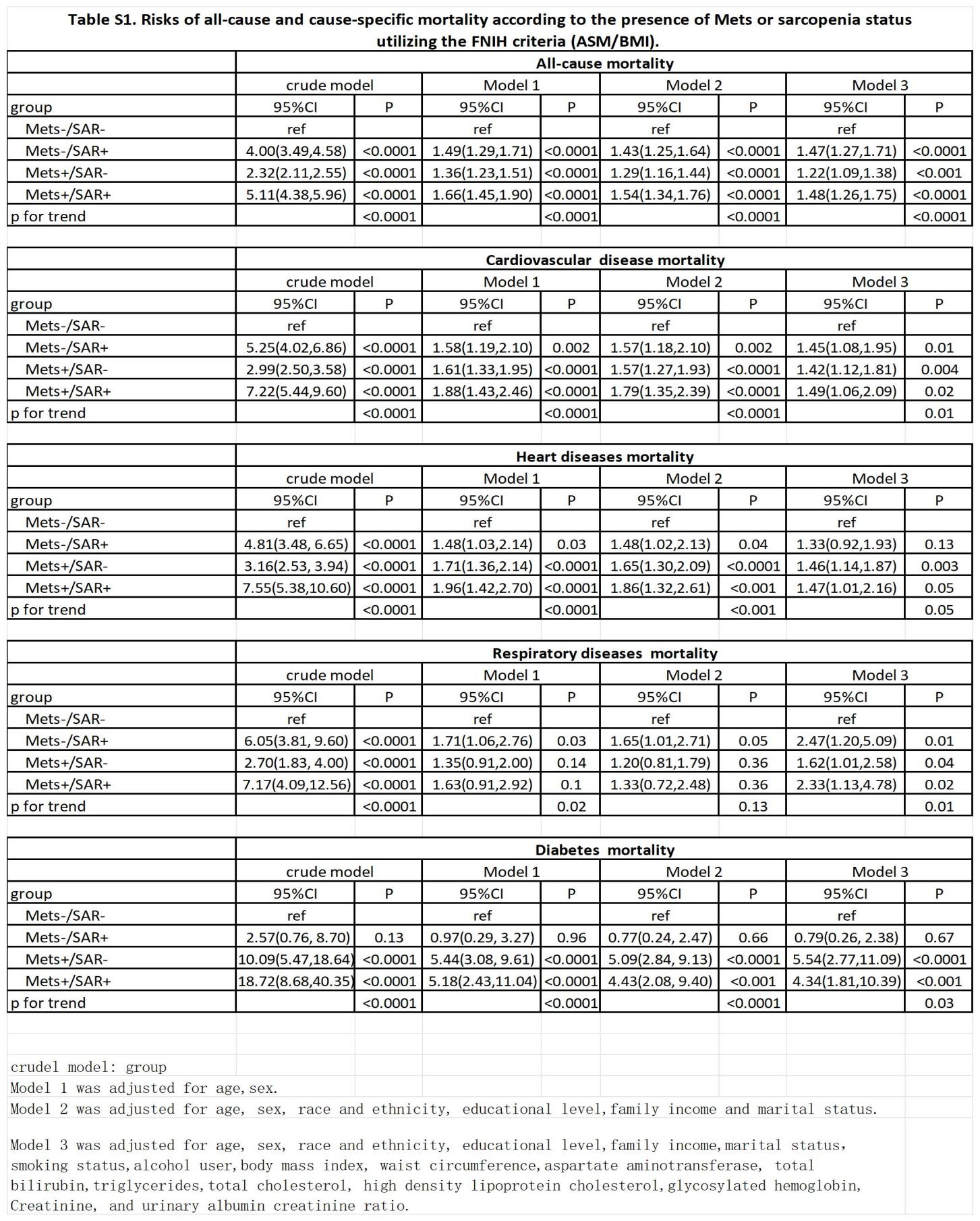


Table S1. Risks of all-cause and cause-specific mortality according to the presence of Mets or sarcopenia status utilizing the FNIH criteria (ASM/BMI).


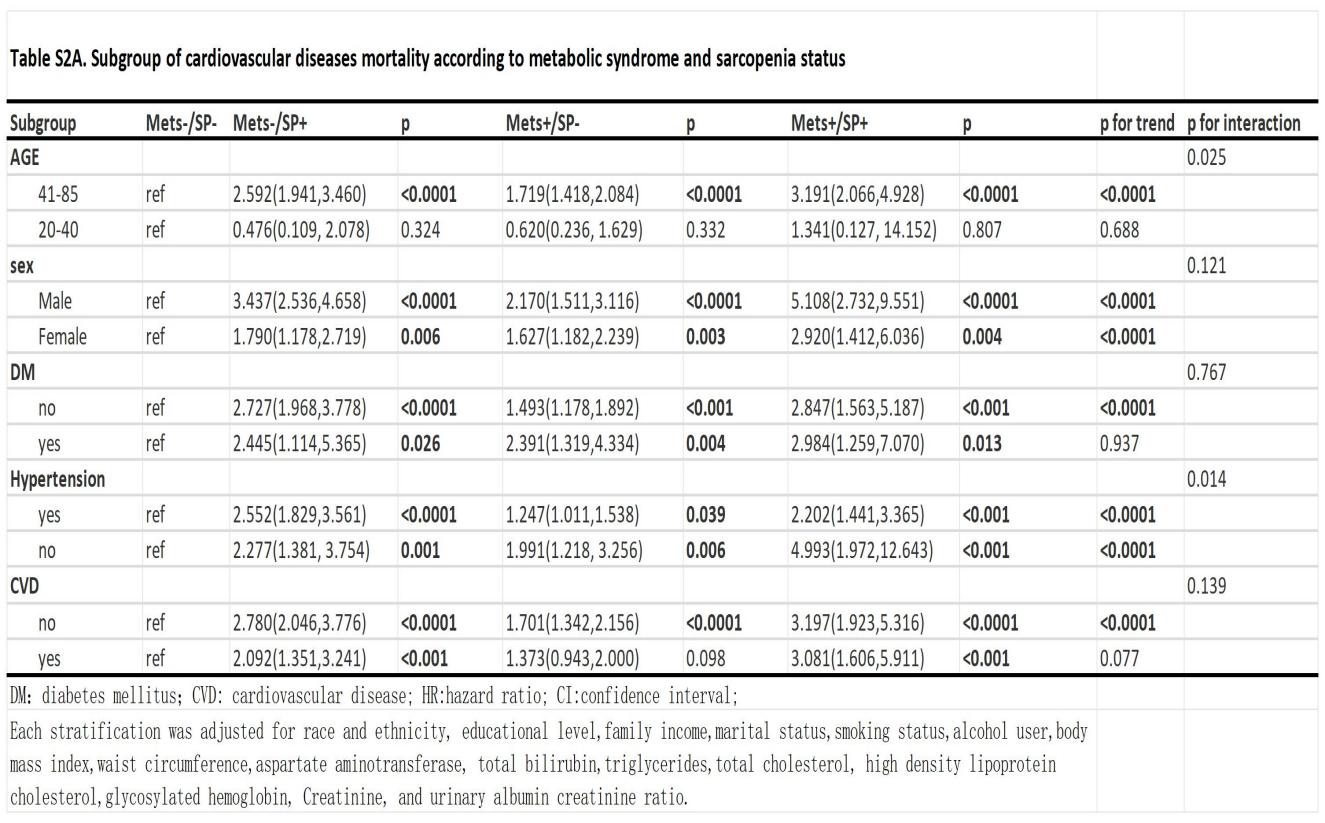


Table S2A. Subgroup of Risks of cardiovascular diseases mortality according to the presence of Mets or sarcopenia status.


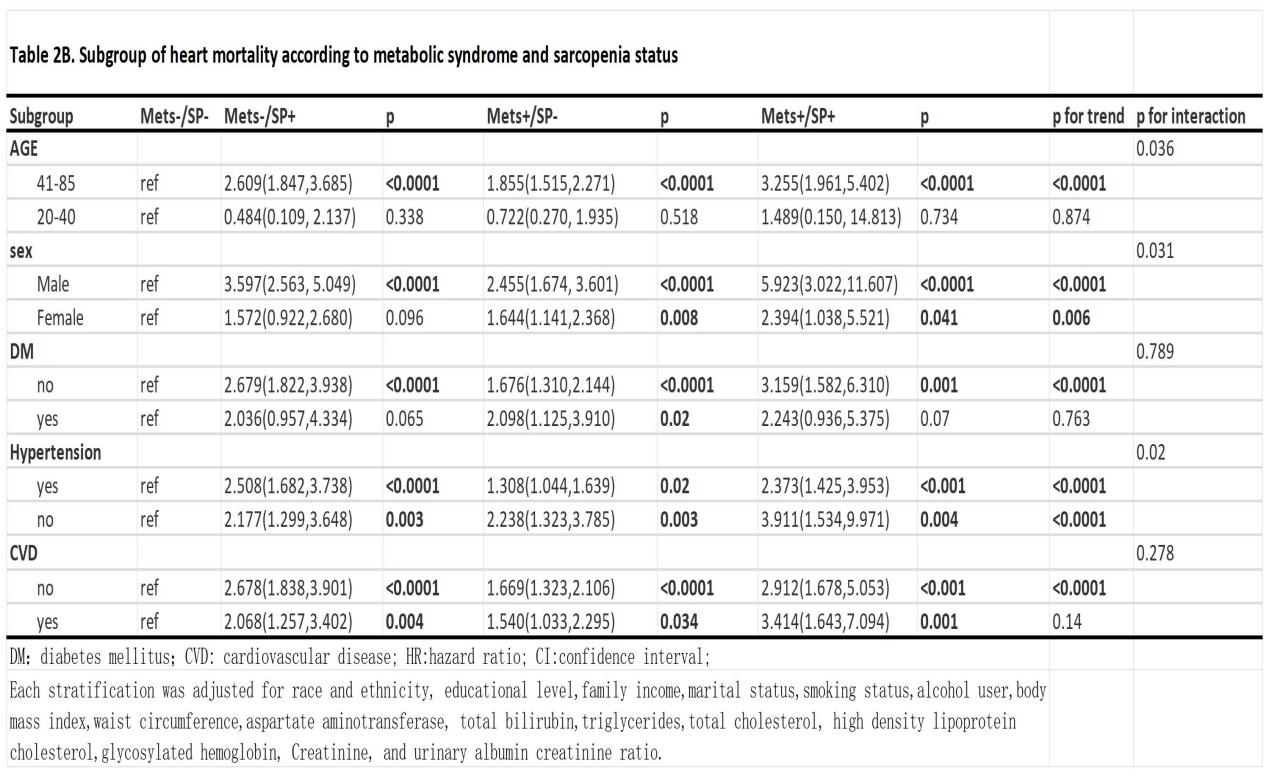


Table S2B.Subgroup of Risks of heart diseases mortality according to the presence of Mets or sarcopenia status.


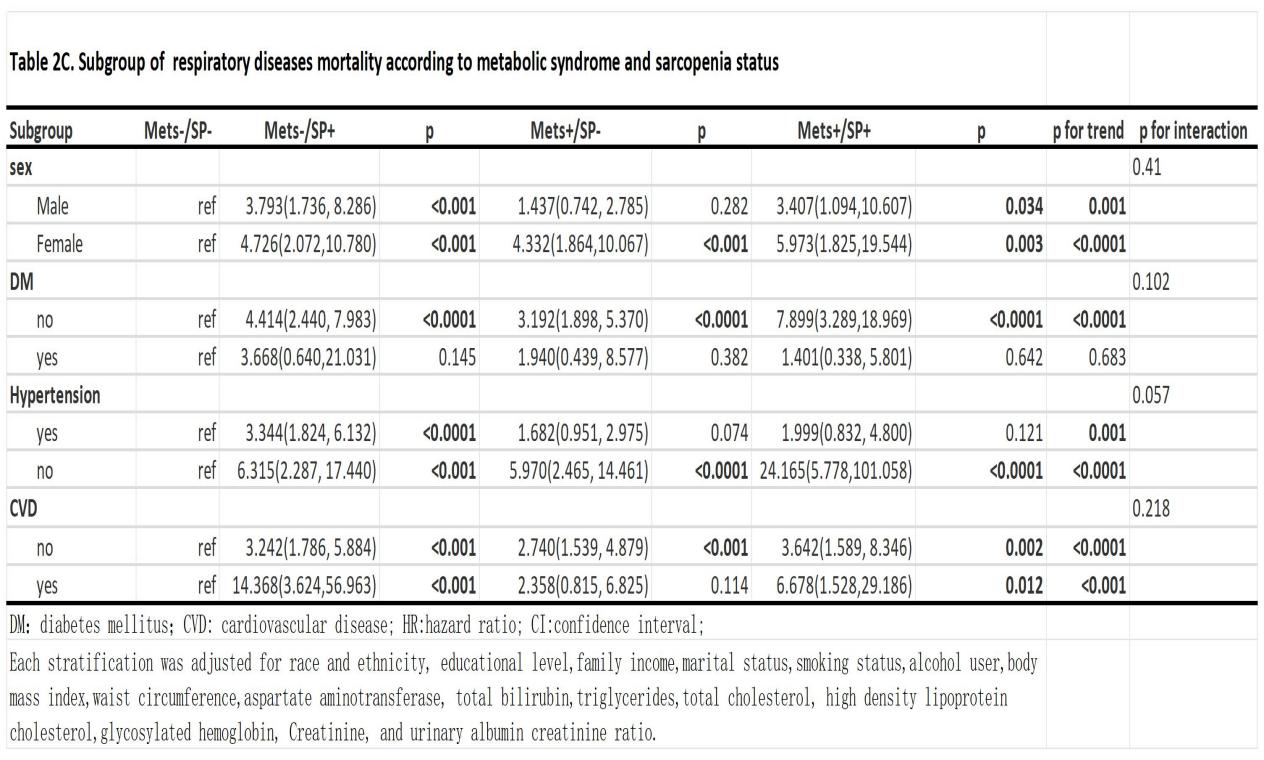


Table S2C.Subgroup of Risks of respiratory diseases mortality according to the presence of Mets or sarcopenia status.


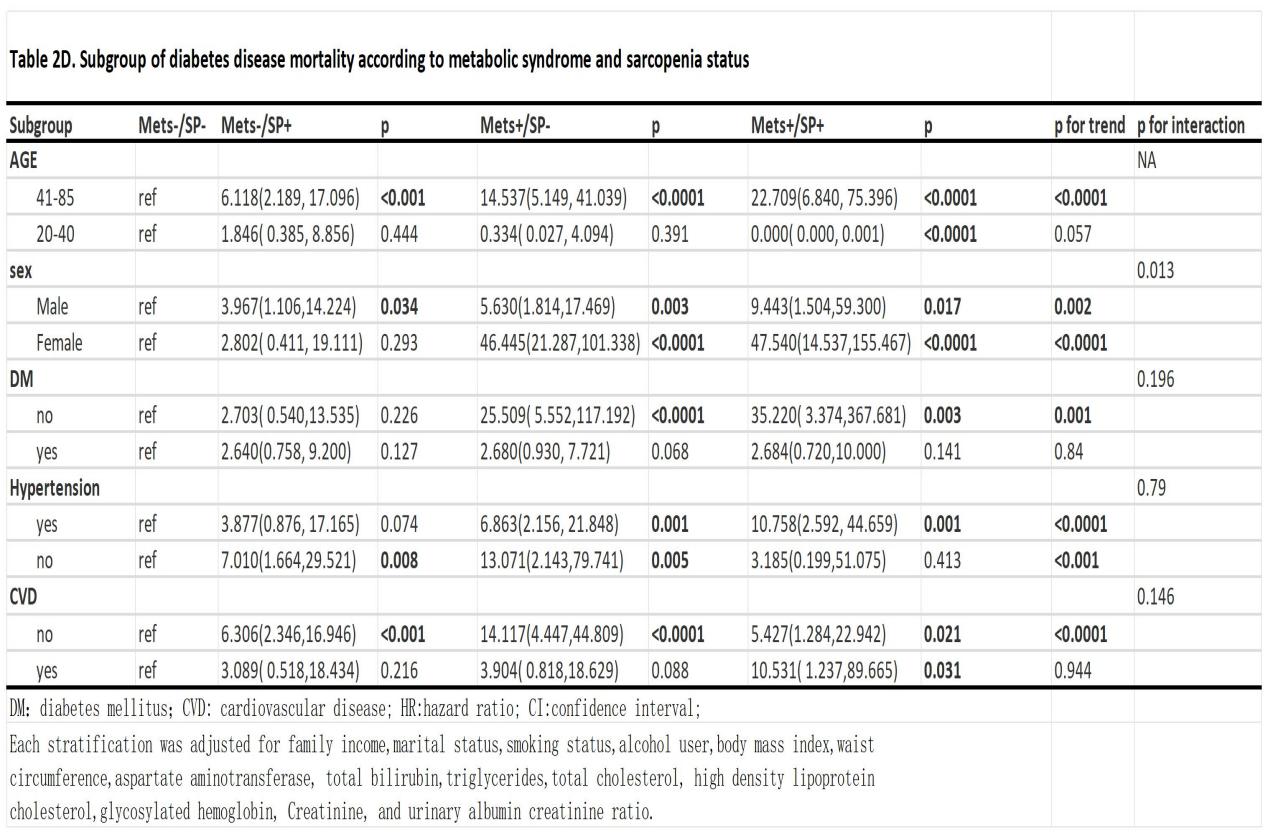


Table S2D.Subgroup of Risks of diabetes mortality according to the presence of Mets or sarcopenia status.


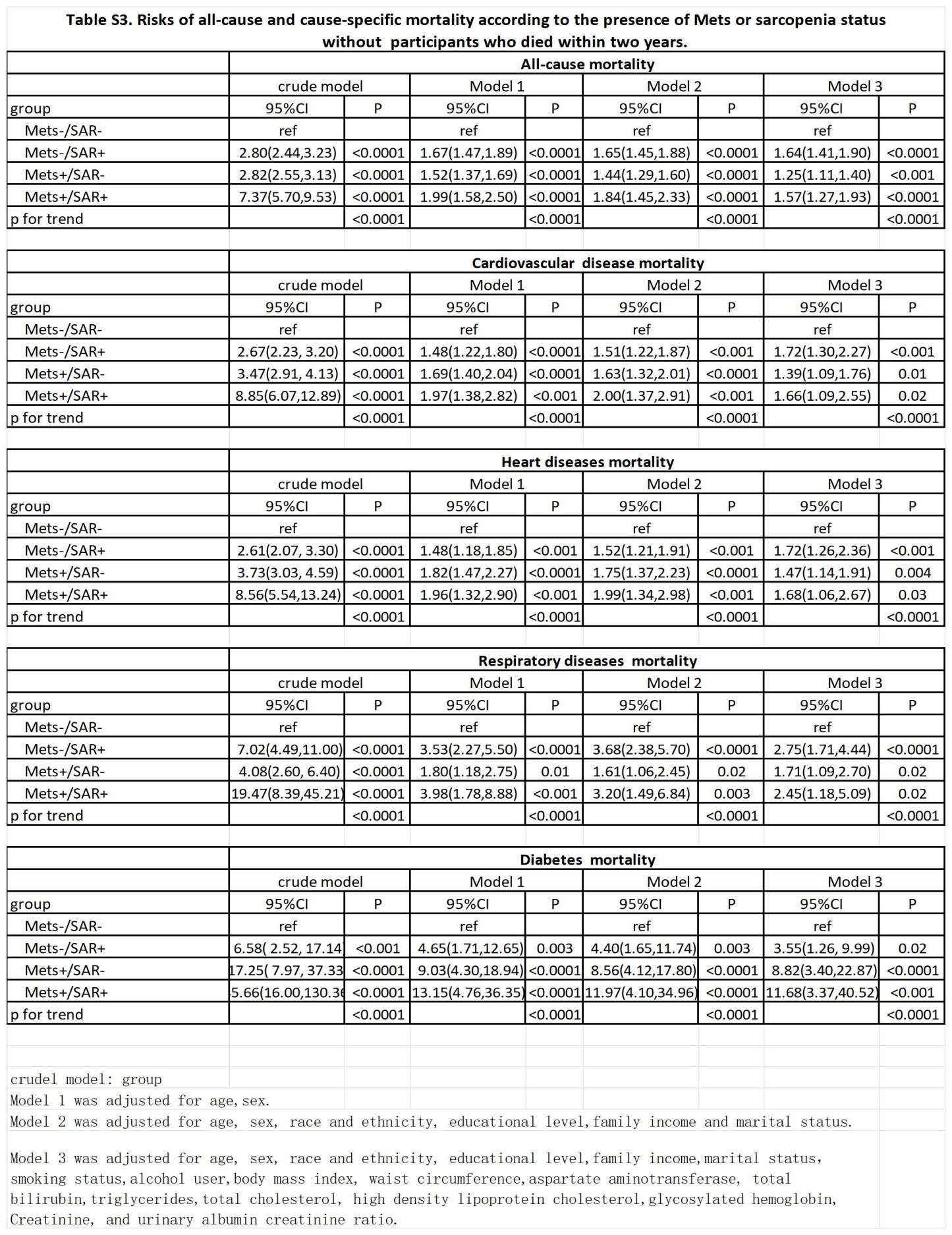


Table S3.Risks of all-cause and cause-specific mortality according to the presence of Mets or sarcopenia status without participants who died within two years.


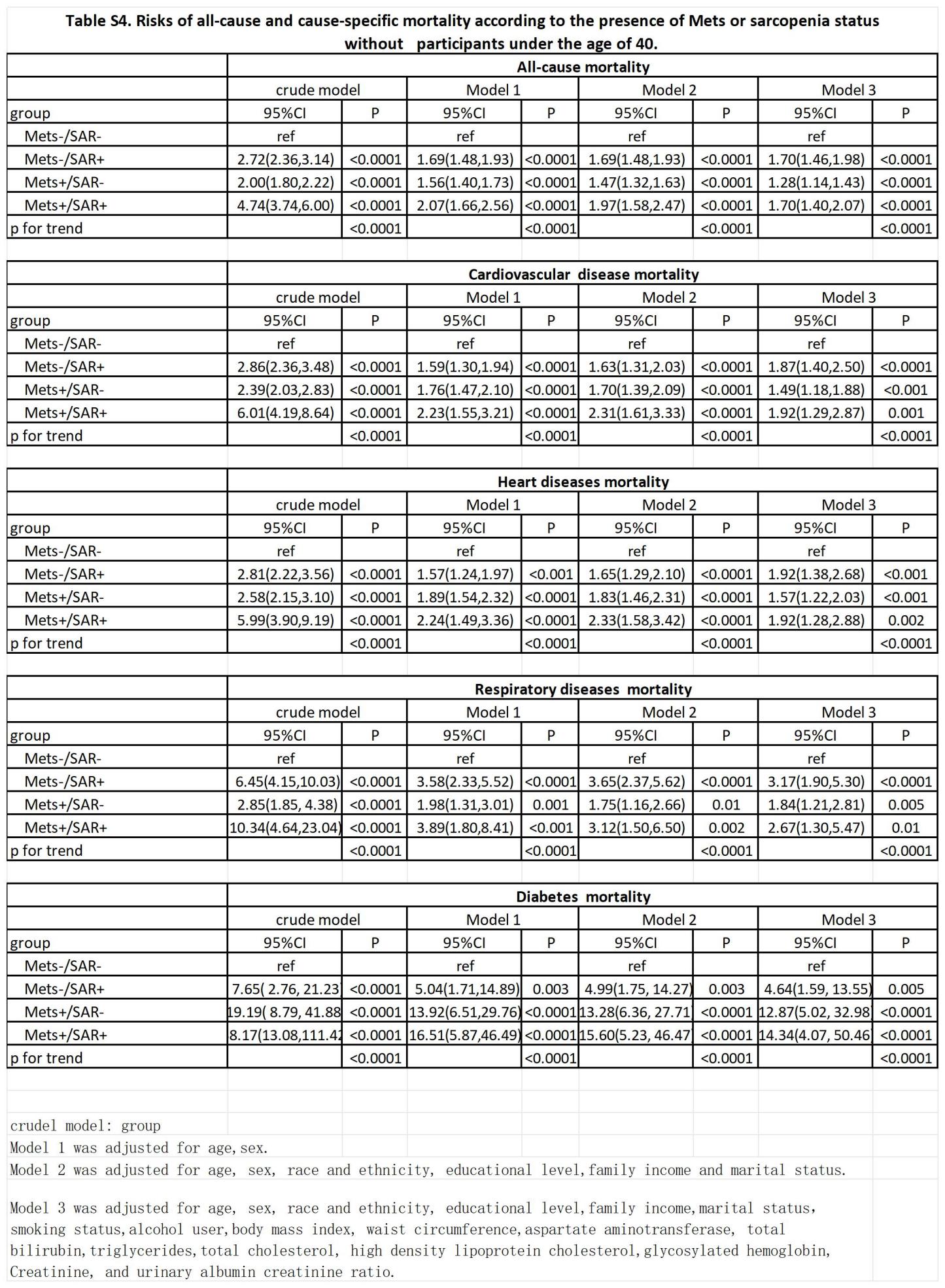


Table S4.Risks of all-cause and cause-specific mortality according to the presence of Mets or sarcopenia status without participants under the age of 40.
